# Multiscale landscape genetics of American marten at their southern range periphery

**DOI:** 10.1038/s41437-020-0295-y

**Published:** 2020-01-28

**Authors:** Cody M. Aylward, James D. Murdoch, C. William Kilpatrick

**Affiliations:** 1 Wildlife and Fisheries Biology Program, Rubenstein School of Environment and Natural Resources, University of Vermont, 81 Carrigan Drive, Burlington, VT 05405 USA; 2 Department of Wildlife, Fish and Conservation Biology, One Shields Avenue, University of California, Davis, CA 95616 USA; 3 Mammalian Ecology and Conservation Unit, Veterinary Genetics Laboratory, University of California, Davis, CA 95616 USA; 4 Department of Biology, University of Vermont, 109 Carrigan Drive, Burlington, VT 05405 USA

**Keywords:** Ecological genetics, Population genetics

## Abstract

American marten (*Martes americana*) are a conservation priority in many forested regions of North America. Populations are fragmented at the southern edge of their distribution due to suboptimal habitat conditions. Facilitating gene flow may improve population resilience through genetic and demographic rescue. We used a multiscale approach to estimate the relationship between genetic connectivity and landscape characteristics among individuals at three scales in the northeastern United States: regional, subregional, and local. We integrated multiple modeling techniques and identified top models based on consensus. Top models were used to parameterize resistance surfaces at each scale, and circuit theory was used to identify potential movement corridors. Regional gene flow was affected by forest cover, elevation, developed land cover, and slope. At subregional and local scales, the effects were site specific and included subsets of temperature, elevation, developed land cover, and slope. Developed land cover significantly affected gene flow at each scale. At finer scales, lack of variance in forest cover may have limited the ability to detect a relationship with gene flow. The effect of slope on gene flow was positive or negative, depending on the site examined. Occupancy probability was a relatively poor predictor, and we caution its use as a proxy for landscape resistance. Our results underscore the importance of replication and multiscale approaches in landscape genetics. Climate warming and landscape conversion may reduce the genetic connectivity of marten populations in the northeastern United States, and represent the primary challenges to marten conservation at the southern periphery of their range.

## Introduction

Habitat conversion for human use has reduced the ranges of many species and fragmented them into smaller isolated patches (Hanski 2011). Population persistence in areas where species are patchily distributed is positively influenced by connectivity and exchange of individuals between patches (Beier and Noss 1998; Haddad et al. 2003; Whiteley et al. 2015). Connectivity reduces the probability of extinction from stochastic events, provides rescue effects following local extirpations, and increases genetic diversity within populations, which can reduce the likelihood of inbreeding depression (Hanski 1997; Quinn et al. 2019). For species affected by habitat fragmentation, identifying and protecting corridors that facilitate dispersal and gene flow between disjunct populations is a conservation priority (Tischendorf and Fahrig 2000).

The American marten (*Martes americana*) is a forest carnivore species that depends on deep snow pack to outcompete larger mesocarnivores (Carroll 2007; Kelly et al. 2009). Martens occur throughout the boreal forests of Canada and Alaska, and were historically widespread in forested regions of the northeastern United States (US) and Great Lakes region (Hagmeier 1956). During the nineteenth and twentieth centuries, anthropogenic land development and unregulated harvest led to widespread population declines, and contracted the southern extent of their range (Hagmeier 1956; Gibilisco 1994).

The southernmost extant population occurs in the northeastern US (O’Brien et al. 2018). Historically, the landscape was almost entirely forested, and the marten population was likely panmictic (Foster et al. 2002). Habitat fragmentation in the nineteenth- to mid-twentieth centuries led to population declines (Gibilisco 1994). As forests have recovered in recent decades, demographic and genetic data suggest that marten populations have also re-expanded (Kelly et al. 2009; Aylward et al. 2019). Nonetheless, the species is regionally considered rare, threatened, or endangered, and maintaining genetic connectivity is a conservation priority (Vermont Wildlife Action Plant Team 2015; New Hampshire Department of Fish and Game 2015). Furthermore, this system affords an opportunity to better understand gene flow dynamics that likely resulted from relatively recent landscape changes.

Gene flow is affected by the extent of landscape connectivity, which is often estimated based on measures of habitat quality like occupancy probability (O’Brien et al. 2006; Stevenson-Holt et al. 2014; Spear et al. 2015; Aylward et al. 2018). However, it is often unclear whether measures of landscape connectivity accurately represent functional connectivity (Tischendorf and Fahrig 2000). Landscape-genetic approaches infer landscape effects on dispersal and migration by examining relationships between genetic differentiation and landscape conditions between sampling locations (Spear et al. 2005; Epps et al. 2007). Identifying landscape characteristics that have positive or negative effects on gene flow can improve management strategies for population connectivity. For example, identifying the impact of major roadways on wildlife population genetic structure contributed to the creation of forested overpass structures that facilitate large mammal gene flow across highways in Banff National Park in Canada (Sawaya et al. 2014).

A common landscape genetics approach involves estimating relationships between genetic distance and an estimated cost of movement between sample locations. This movement cost is often calculated by using a resistance surface, a gridded representation of the landscape in which each cell value represents the degree to which the landscape conditions inhibit dispersal (Spear et al. 2015). Next, techniques like circuit theory can be applied to estimate the likelihood of dispersal and the most probable movement corridors between two points (McRae 2006). Resistance surfaces are challenging to parameterize, often relying on *a priori* assignment of resistance values to certain landscape characteristics (Spear et al. 2015). One approach, called causal modeling, limits biases associated with *a priori* resistance assignments by allowing the genetic data to determine the optimal parameterization scheme (Cushman et al. 2006). Causal modeling involves testing a wide range of resistance values for each landscape variable, and identifying the optimal parameterization based on correlation coefficients or information criteria (Smouse et al. 1986; Legendre et al. 1994; Burnham and Anderson 2002).

In this study, we used a hierarchical approach to parameterize models that predict how landscape conditions affect genetic connectivity of American marten populations in the northeastern US. The landscape conditions that facilitate or inhibit long-distance dispersal between subpopulations may differ from those that facilitate or inhibit local dispersal within subpopulations (Parks et al. 2013). Furthermore, observed landscape-genetic relationships may vary in different parts of a species’ range (Short Bull et al. 2011). Therefore, we estimated landscape-genetic relationships across the entire study area (“regional” scale), among groups of subpopulations (“subregional” scale), and within subpopulations (“local” scale). We used multiple analytical approaches to construct candidate models and verify their predictive power. We also identified potential dispersal corridors at each spatial scale.

## Methods

### Study area

**Fig. 1:**
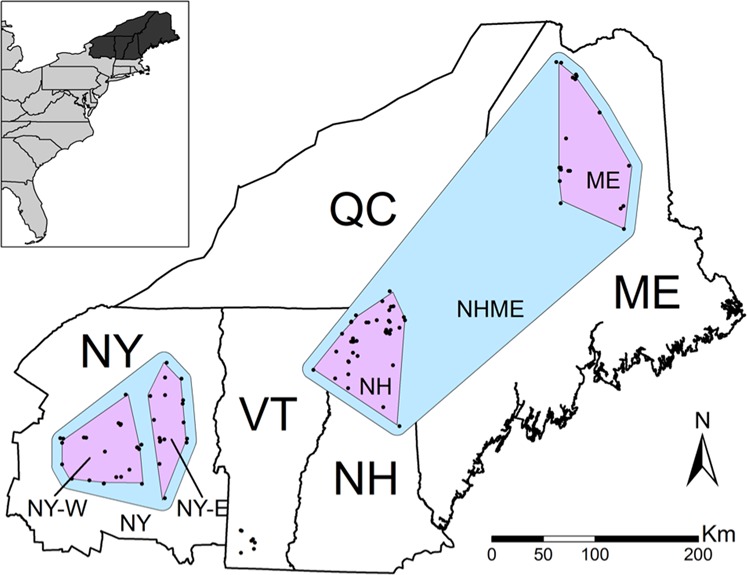
The study area includes the US states of Maine (ME), New Hampshire (NH), and Vermont (VT), the Adirondack Mountain region of New York (NY), and part of the Canadian province of Quebec (QC) south of the St. Lawrence River. Dots represent individual marten sample locations used in this study. Blue polygons show the extent of subregional study areas, which were determined from broad-scale genetic population clusters (Aylward et al. 2019). Purple polygons show the extent of local study areas, which were determined by finer-scale population genetic clustering (Aylward et al. 2019). The inset shows the location of the study area (dark shading) in relation to the Atlantic coast of the United States. Samples in southern Vermont were not used for model development because of human-mediated gene flow from a reintroduction attempt. However, this location was used in corridor modeling due to the importance of identifying corridors for genetic connectivity to this population.

The study area included the US states of Vermont, New Hampshire (NH), and Maine (ME), the Adirondack Mountain region of New York (NY), and part of the Canadian province of Quebec, south of the St. Lawrence River (total area = 220,132 km^2^, Fig. 1). This area occurred along the southern limit of marten distribution. The study area was defined by regions harboring marten populations in the northeastern US and areas most likely to be used for long-distance dispersal between populations. The region is characterized by historically widespread forests that experienced significant fragmentation following European colonization (Foster et al. 2002). Forests have been in recovery in the region since the mid-1900s (Foster et al. 2002). Today, the study area is ~80% forested land cover, and more specifically, 20% spruce–fir forest, which may be the preferred cover type for martens (Bowman and Robitaille 1997; Godbout and Ouellet 2010). Demographics of forest mammals in the region are believed to have followed a similar trajectory, declining through the 1800s and recovering within the past several decades (Foster et al. 2002; Giblisco 1994; Hapeman et al. 2011; Aylward et al. 2019).

### Genetic data

Genetic data used in this study were a subset of microsatellite data previously used to estimate genetic structure in the northeastern US (Aylward et al. 2019). Genetic material was obtained from tissue samples of animals collected by trappers in NY and ME, where martens are legally harvested, and incidental take by trappers and road kill in Vermont and NH, where martens are endangered and threatened, respectively. Previous estimates suggest hierarchical genetic structure within the region (Aylward et al. 2019). At a broad scale, two genetic clusters were present, which are referred to as “subregional” sites in this study: (1) NY and (2) New England (NHME). At a fine scale, five subpopulations were present: (1) ME, (2) New Hampshire and north-eastern Vermont, (3) southern Vermont (VT-S), (4) eastern New York (NY-E), and (5) western New York (NY-W). These fine-scale subpopulations are referred to as “local” sites in this study. We removed the subpopulation in VT-S from this analysis, as it was likely reintroduced from ME (Aylward et al. 2019), and translocations result in genetic patterns that are not indicative of natural processes (Colella et al. 2019). Furthermore, we removed any individual whose township locality could not be determined. The dataset for this analysis included ten microsatellite loci from 102 individuals from the four remaining subpopulations. We used these data to produce a novel analysis of landscape effects on the observed genetic distances between individuals.

Individual-based genetic distance was estimated in the R package “Gstudio” (Dyer 2012; R Core Team 2018) using the dist_euclidean function. Euclidean genetic distance has been shown to perform well in individual-based landscape genetics analysis (Shirk et al. 2018). Sample locations were obtained at the township level. Although precise GPS locations would be preferable, township-level data were the finest scale available for the majority of samples. To facilitate an individual-based approach, we assigned a location for each sample within its given township. Locations were randomly selected 1–3 km from the geographic center of their township.

### Landscape data

Spatial data for landscape variables were obtained from public sources and scaled to the raster resolution of the coarsest dataset (800 × 800 m, Supplementary Information I). Based on previous research of marten habitat use, we considered seven landscape variables: (1) forest land cover, (2) spruce–fir land cover, (3) developed land cover, (4) elevation, (5) winter (Nov–Mar) temperature, (6) road density, and (7) slope (Bowman and Robitaille 1997; Kelly et al. 2009; Godbout and Ouellet 2010). We also tested the performance of estimated occupancy probability as a predictor using a model derived from expert-opinion data in the northeastern US (Aylward et al. 2018). Incongruence of spatial data across state or country boundaries limited our ability to include other desirable variables, such as tree canopy cover.

For each landscape variable, we constructed resistance surfaces for a range of maximum resistance (*R*_max_) values ranging from *R*_max_ = 2–500 (Roffler et al. 2016; Supplementary Information II). Landscape variables were coded such that features hypothesized to reduce gene flow were assigned higher values in the resistance surface. Variables hypothesized to be positively related to gene flow included forest land cover, spruce–fir land cover, elevation, and estimated occupancy. Therefore, these variables were reverse transformed to create resistance surfaces (e.g., 100% forested cover = 1 and 0% forested cover = *R*_max_). Landscape variables hypothesized to have a negative relationship with gene flow included winter temperature, developed land cover, road density, and slope.

We then estimated the resistance distance between each individual using Circuitscape (McRae et al. 2008). The resistance distance based on a model of isolation by distance (IBD) was estimated by creating a null resistance surface in which the resistance value of each cell was 1, which is considered the appropriate null model for Circuitscape-based analyses (Roffler et al. 2016; Tucker et al. 2017).

### Resistance surface parameterization

The optimal *R*_max_ for each landscape variable was determined by estimating the relationship between genetic distance and landscape resistance distance for univariate models. For analyses with underlying population substructure (regional and subregional scale), we used maximum-likelihood population effects (MLPE) models constructed using lme4 (Bates et al. 2015) to estimate landscape-genetic relationships (Clarke et al. 2002). For analyses within a single population (local scale), we conducted partial Mantel tests (Smouse et al. 1986) using Ecodist (Goslee and Urban 2007). MLPE models outperform Mantel and other regression methods when population structure is present, whereas Mantel methods perform well in the absence of population structure (Franckowiak et al. 2017; Row et al. 2017; Shirk et al. 2018). The optimal *R*_max_ for each landscape variable was determined by the *R*_max_ with the lowest AICc in MLPE models and highest *R*^2^ in Mantel models (Shirk et al. 2018).

Multivariate models were then constructed by combining subsets of landscape variables. Only the optimal *R*_max_ was used for each landscape variable in multivariate models. We removed geographic distance from each landscape variable to isolate the impact of the landscape variable on landscape resistance (Tucker et al. 2017). Regional and subregional analyses used MLPE modeling for multivariate models, and local analyses used multiple regression of distance matrices (MRDM; Legendre et al. 1994) in Ecodist. We identified several criteria to ensure that models contained informative and uncorrelated variables. First, we restricted multivariate models to include no more than one variable from each of the following categories: forest characteristics (forest land cover; spruce–fir land cover), anthropogenic land covers (developed land cover; road density), and climatic variables (elevation; winter temperature). Estimated occupancy was not included in multivariate analyses, serving as an alternative hypothesis. Next, we excluded all models with significant multicollinearity (one or more variables with a variance inflation factor, VIF > 5) or uninformative landscape variables (*β* coefficient 95% confidence intervals included 0).

Multivariate models were ranked by AICc, which had strong concordance with *R*^2^ in MRDM models. For each study site at each scale, the top models that contributed to 99% of the AICc weight were identified. One common approach is to model-average top scoring models (Symonds and Moussalli 2011), however, this could reintroduce predictor variables that were previously excluded from inclusion in the same model. As an alternative, we conducted a commonality analysis (CA; e.g., Prunier et al. 2015) in the R program “yhat” (Nimon et al. 2013) to select a single model from the top AICc model set to represent the resistance surface for each study site. We computed structure coefficients (rs) for each variable, an estimate of the amount of variance in the dependent variable explained by each predictor irrespective of collinearity among predictors (Prunier et al. 2015). We eliminated any model with an independent variable whose rs did not differ significantly from 0. We then used CA to estimate the amount of variance in genetic distance explained uniquely by each independent variable (*U*) and shared among other predictors (*C*), which sum to the total explanatory contribution of the variable (*T*) (Nimon and Oswald 2013; Prunier et al. 2015). The model with the set of predictors that contributed to the greatest amount of variance in genetic distance (based on the summed *T* of independent variables) was chosen to parameterize the resistance surface for each study site. Resistance surface parameterization was conducted based on *β* weights of predictor variables in the resistance surface model.

### Corridor mapping

Resistance surfaces for each site at each scale were created in Raster Calculator in ArcGIS 10 (ESRI, Redlands, California, USA) by calculating a dot product of β-coefficients in the model expression with the respective landscape variable values. Resistance surface rasters were scaled 1–100 for use in Circuitscape (McRae et al. 2008). At the regional scale, where conservation objectives are often to identify long-distance corridors between isolated subpopulations, we used Linkage Mapper (McRae and Kavanagh 2011) to estimate corridors. Focal nodes were represented by the minimum convex polygon of marten locations for each local population. At the subregional and local scales, where practical corridor end points are less clear, we used Circuitscape to estimate current density, as a proxy for probability of gene flow, throughout each study site. To limit focal node attraction bias, according to recommendations by Koen et al. (2014a), we buffered each site by ~20% the width of the study site and placed 30 focal nodes evenly spaced along the perimeter of the buffer.

## Results

### Regional

Sixty-five models were fitted for the regional univariate analysis: eight *R*_max_ values for seven landscape variables and occupancy probability plus the null IBD model (Supplementary Information II). Selected *R*_max_ values ranged from the highest value tested (*R*_max_ = 500; developed land cover) to the lowest value tested (*R*_max_ = 2; slope; Supplementary Information II). The range of *R*_max_ values tested in our analysis is considered extensive based on previously published literature (Roffler et al. 2016; Tucker et al. 2017), and we considered it unnecessary to expand the range of values tested.

After identifying the optimal *R*_max_ for each landscape variable, 55 multivariate models were fitted (Supplementary Information III). After removing models with significant VIF and uninformative parameters, seven models contributed to 99% of the AICc weight (Table 1). Variables included in these models were forest land cover, spruce–fir land cover, elevation, winter temperature, developed land cover, and slope. The For + Elev + Dev + Slope model had the greatest explanatory power (*T* = 17.9; Table 1), and was used to create the resistance surface.

**Table 1:** Top-ranking landscape resistance models in each study site ranked by AICc and ΔAICc.

Study site	Model	AICc	ΔAICc	AICc W	AICc Wc	*R*^2^	*p*	*T*
Regional								
NE	*For + Elev + Dev + Slope	3530.24	0.000	0.407	0.407	–	–	17.9
	SF + Dev + Slope	3530.57	0.334	0.344	0.750	–	–	10.6
	For + Temp + Dev + Slope	3533.12	2.890	0.096	0.846	–	–	16.9
	Elev + Dev + Slope	3533.38	3.141	0.085	0.931	–	–	11.0
	For + Elev + Dev	3535.13	4.895	0.035	0.966	–	–	16.8
	Elev + Dev	3535.99	5.750	0.023	0.989	–	–	9.9
	SF + Dev	3538.14	7.902	0.008	0.997	–	–	9.6
Subregional								
NY	*Dev + Slope	607.53	0.000	0.335	0.335	–	–	4.2
	SF + Dev + Slope	607.65	0.115	0.316	0.651	–	–	4.6^†^
	Elev + Dev + Slope	608.03	0.503	0.261	0.912	–	–	4.3^†^
	Elev + Roads + Slope	611.14	3.611	0.055	0.967	–	–	3.5
	SF + Roads + Slope	612.28	4.743	0.031	0.998	–	–	4.4^†^
NHME	SF + Roads + Slope	1158.67	0.000	0.690	0.690	–	–	5.7
	SF + Dev + Slope	1162.94	4.271	0.082	0.772	–	–	5.7
	For + Elev + Dev + Slope	1163.37	4.705	0.066	0.837	–	–	10.2^†^
	Dev + Slope	1163.50	4.836	0.061	0.899	–	–	1.1
	SF + Slope	1163.56	4.896	0.060	0.958	–	–	5.4
	*Elev + Dev + Slope	1165.91	7.244	0.018	0.977	–	–	6.6
	Temp + Slope	1167.22	8.557	0.010	0.986	–	–	3.3
	Elev + Slope	1168.46	9.788	0.005	0.992	–	–	6.2
Local								
NY-W	*Dev + Slope	235.04	0.000	0.998	0.998	0.117	0.030	10.0
NH	Temp + Roads	440.32	0.000	0.899	0.899	0.065	<0.001	5.3
	*Temp + Slope	444.94	4.614	0.090	0.988	0.060	<0.001	8.0
	Temp	449.14	8.817	0.011	0.999	0.053	<0.001	5.2

Top models are those whose AICc weight (AICc W) contributes to 99% of the cumulative AICc weight (AICc Wc) in the model set. For local study areas, *R*^2^ and *p* values from MRDM are reported. Covariates include forest land cover (For), spruce–fir land cover (SF), winter temperature (Temp), elevation (Elev), developed land cover (Dev), road density (Roads), and slope (Slope). Asterisk indicates the model from each study site that was chosen to create a resistance surface for downstream analyses, based on commonality analysis assessments of variables’ contribution to the total variance in genetic distance (*T*). Models that had the greatest *T* in the study area, but contained variables whose structure coefficient (rs) did not differ from zero, have *T* estimates marked by a cross (†).

### Subregional

In each subregion, 65 models were fitted for univariate analyses, and 55 models were fitted for multivariate analyses, coinciding with the model set tested in the regional analysis. In the NY subregion, most landscape variables had an optimal *R*_max_ of 2, with the exception of forest land cover (*R*_max_ = 50), developed land cover (*R*_max_ = 500), and slope (*R*_max_ = 10; Supplementary Information II). After removing models with high VIF and uninformative landscape variables, five models contributed to 99% of the AICc weight (Table 1; Supplementary Information III). Variables included in these models were spruce–fir land cover, elevation, developed land cover, roads, and slope. The SF + Dev + Slope model had the greatest explanatory power (*T* = 4.6; Table 1), but spruce–fir land cover had nonsignificant rs. The Dev + Slope model (*T* = 4.2) was selected to create the resistance surface.

In the northern New England (NHME) subregion, most landscape variables had an optimal *R*_max_ of 2, with the exception of developed land cover (*R*_max_ = 10) and slope (*R*_max_ = 10, Supplementary Information II). After removing models with high VIF or uninformative landscape variables, eight models contributed to 99% of the AICc weight (Table 1; Supplementary Information III). All landscape variables tested were included in this set of models (Table 1). The For + Elev + Dev + Slope model had the greatest explanatory power (*T* = 10.2) but forest had nonsignificant rs. The Elev + Dev + Slope model was the second-most explanatory (*T* = 6.6) and was used to create the resistance surface.

### Local

Each local analysis included the same set of 65 univariate and 55 multivariate models fitted in the regional and subregional analyses. In NY-W, optimal *R*_max_ = 500 for all landscape variables except forest land cover (*R*_max_ = 2), spruce–fir land cover (*R*_max_ = 2), and developed land cover (*R*_max_ = 50; Supplementary Information II). After removing models with high VIF or uninformative landscape variables, one model (Dev + Slope) contributed to 99% of the AICc weight (Table 1; Supplementary Information III). CA revealed developed land cover and slope as meaningful predictors, and the Dev + Slope model (*T* = 10.0) was used to create the resistance surface.

In NH, the optimal *R*_max_ was 2 for all landscape variables, except developed land cover (*R*_max_ = 5), forest land cover (*R*_max_ = 200), and road density (*R*_max_ = 500; Supplementary Information II). After removing models with high VIF or uninformative landscape variables, three models contributed to 99% of the AICc weight (Table 1; Supplementary Information III). Landscape variables in these models included winter temperature, road density, and slope (Table 1). The Temp + Slope model had the greatest explanatory power (*T* = 8.0) and was used to create the resistance surface.

In NY-E and ME, all models included uninformative landscape variables. These sites had the smallest sample sizes (*n* = 16 for NY-E and *n* = 18 for ME), which may be responsible for low statistical power in the study areas.

### Corridors

**Fig. 2:**
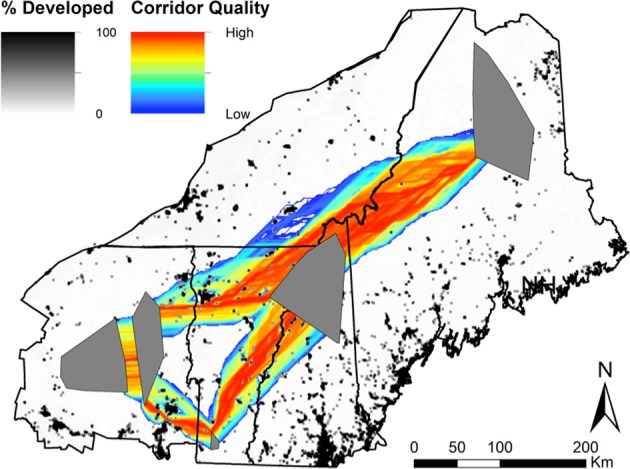
Regional corridors displayed over a raster map of developed land cover in the study area. Gray polygons show the location of focal areas, which were the five fine-scale genetic clusters identified based on microsatellite data in a previous study (Aylward et al. 2019). Within corridors, hot colors (red) indicate higher corridor quality and cool colors (blues) indicate lower quality corridors. Corridors are cut off at resistance cost of 10 cost-weighted kilometers. Apparent holes within corridors occur where small patches of developed land create high landscape resistance and are completely avoided.

At the regional scale, the resistance surface included the effects of forest land cover (*U* = 0.4) and elevation (*U* = 1.2), both negatively correlated with landscape resistance, and developed land cover (*U* = 1.2) and slope (*U* = 0.1), both positively correlated with landscape resistance (Table 2). Corridors connected core populations via relatively straight paths with notable avoidance of developed land cover (Fig. 2).

**Fig. 3:**
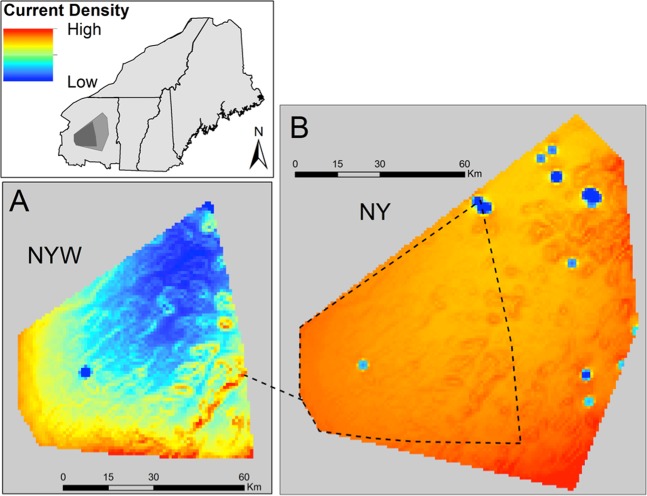
Current density estimates based on landscape genetics models in subregional and local sites in New York. Current density estimated across the NY-W local site (**a**) and NY subregion (**b**) using Circuitscape, based on resistance surfaces parameterized by landscape genetics models. Dark shading on the inset indicates the extent of the NY-W local site and intermediate shading indicates the extent of the NY subregion. Current density serves as a proxy for probability of gene flow such that high current density areas are more likely to be used as corridors for movement. In both sites, landscape resistance was predicted by developed land cover (positively correlated with landscape resistance) and slope (negative).

**Fig. 4:**
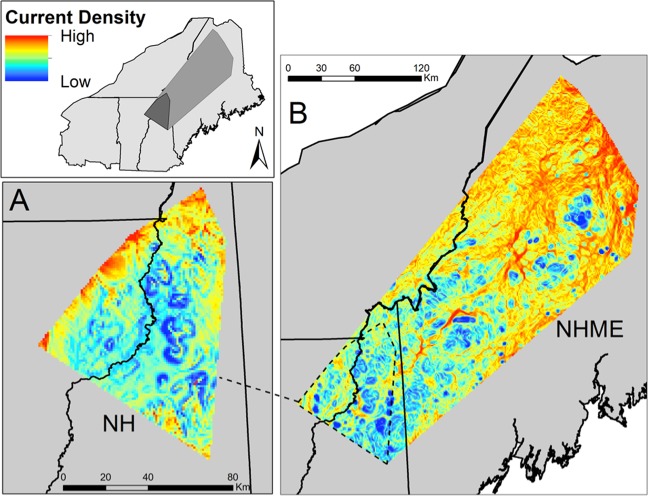
Current density estimates based on landscape genetics models in subregional and local sites in northern New England. Current density estimated across the NH local site (**a**) and NHME subregion (**b**) in Circuitscape, based on resistance surfaces parameterized by landscape genetics models. Dark shading on the inset indicates the extent of the NH local site and intermediate shading indicates the extent of the NHME subregion. Current density serves as a proxy for probability of gene flow such that high current density areas are more likely to be used as corridors for movement. In the NHME subregional site, landscape resistance was predicted by elevation (negatively correlated with landscape resistance), developed land cover (positive), and slope (positive). In the NH local site, landscape resistance was predicted by winter temperature (positive) and slope (positive).

In the NY subregion, the resistance surface included the effects of developed land cover (positive, *U* = 2.1) and slope (Table 2). Current density (proxy for probability of gene flow) was relatively high throughout the study area, with notable small patches of low current density in developed areas (Fig. 3b). In the NHME subregion, the resistance surface included the effects of elevation (negative, *U* = 0.7), developed land cover (positive, *U* = 0.6), and slope (Table 2). A complex mosaic of current densities occurred throughout NHME (Fig. 4b). In general, current densities were highest in the northern parts of the study site.

In the NY-W local site, the resistance surface included the effects of developed land cover (positive, *U* = 7.7) and slope (Table 2). High current densities occurred in the steep ridgelines in the south and east parts of the site (Fig. 3a). In the NH local site, the resistance surface included the effects of winter temperature (positive; *U* = 3.3) and slope (Table 2). High current densities occurred in the highlands in the north and northwest of the study area, while low current densities occurred on steep slopes and in the warmer lowlands in the center of the study site (Fig. 4a).

## Discussion

### Regional scale

The resistance surface at the regional scale included effects from all four categories of landscape variables examined (forest characteristics, climate variables, anthropogenic land cover, and slope). Corridors were relatively nonspecific with the exception of movement barriers where developed land cover occurred. Contrary to previous corridor estimates based on occupancy (Aylward et al. 2018), the central and northern Green Mountains of Vermont were not considered an important corridor between populations in VT-S, NH, and NY. Martens are considered extraordinarily successful dispersers, exhibiting low levels of genetic distance per geographic distance compared with other mammals of similar or larger body size (Kyle and Strobeck 2003). The misalignment of corridors identified from occupancy-based and landscape genetics analyses may be due to greater flexibility in habitat use during dispersal than residency, as has been observed in other carnivore species (Palomares et al. 2000).

### Subregional scale

Both subregional sites included developed land cover and slope as effects in their resistance surfaces. In NY these were the only two variables in the resistance surface. In NHME, elevation was also included. Landscape resistance was associated with low elevations in NHME and high developed land cover in both sites. Interestingly, the correlation between landscape resistance and slope was positive in NHME and negative in NY. This pattern was observed across all top models that included slope in both NHME and NY subregions (data not shown). Slope may facilitate gene flow due to spatial correlation with elevation or low temperatures in NY. However, including elevation or temperature in NY models (i.e., Elev + Dev + Slope or Temp + Dev + Slope models) did not change the sign or significance of the effect of slope in the NY subregion. In other species, steep slopes can be positively correlated with genetic distance due to increased energetic cost of travel (Funk et al. 2005, Spear et al. 2005) or negatively correlated with genetic distance due to “escape habitat” from larger predators or better opportunities for vigilance (Epps et al. 2007; Portanier et al. 2018). The site-dependent reversal of the relationship between slope and genetic distance in our study shows that sampling site selection can significantly alter landscape-genetic inferences.

### Local scale

The NY-W resistance surface included the same landscape effects (Dev + Slope) as the NY subregion. Steep areas in the south of NY-W were identified as having the highest current density, in agreement with results from the subregional scale. Winter temperature and slope were included in the NH resistance surface. Similar to the subregional results, slope was positively associated with landscape resistance in NH despite having a negative relationship in NY-W. At the local level, the suite of landscape variables in top-performing landscape genetics models was site dependent. This result underscores that results obtained from landscape genetics modeling may not apply outside the specific study area (Short Bull et al. 2011; Castillo et al. 2016).

### Scale dependence of landscape-genetic relationships

Developed land cover negatively affected gene flow in all sites at all scales. Forest land cover was present in the regional resistance surface but was absent from resistance surfaces at smaller scales. The smaller study areas are constrained to areas where martens occur, thus contain comparatively little unforested land (<12% for all local study areas, 20% in the regional study area; Supplementary Information IV). The lack of an observed effect of forest characteristics on gene flow at finer scales is probably not biological, and may be a product of the lack of variance in forest cover at finer-scale sites. This is an important consideration for future landscape genetics studies, as the lack of an observed statistical effect may be more related to sampling decisions than to biological relationships between landscape conditions and gene flow.

We expected the effect of climate variables to be the strongest in NY sites, which experience higher temperatures that would be more likely to constrain marten habitat use and gene flow (mean winter temperature [°C]: NY = 0.085, NHME = −1.22, Supplementary Information IV). However, NY and NY-W were the only sites in which a climate variable (elevation/temperature) did not play a role in the resistance surface. The NY sites have relatively low variance in elevation and winter temperature (Supplementary Information IV). Consequently, the lack of an observed effect of climate on gene flow in NY may be nonbiological; these sites may simply lack adequate spatial heterogeneity in temperature and elevation to produce a detectable effect.

Occupancy probability was not a strong predictor of genetic connectivity at any scale compared with multivariate models parameterized by genetic distance. Habitat suitability or occupancy models are often used as a proxy for landscape permeability (O’Brien et al. 2006; Stevenson-Holt et al. 2014; Spear et al. 2015; Aylward et al. 2018). Our results caution that occupancy does not necessarily predict genetic connectivity in a landscape genetics framework, perhaps due to animals exhibiting greater flexibility in habitat use while transient than when selecting home ranges (Mateo-Sanchez et al. 2015). Occupancy-based predictions of genetic connectivity may be more suitable for species with highly restrictive habitat use or low mobility (Wang et al. 2008).

### Context

Genetic connectivity of marten populations in the interior of their range is better predicted by IBD than additional landscape effects (Kyle et al. 2000; Kyle and Strobeck 2003; Broquet et al. 2006; Koen et al. 2012). In our study area at the southern periphery of marten range, connectivity was better described by models that included landscape covariates (Supplementary Information V). In particular, at least one site at each scale examined included a climate-related variable and developed land cover in the top-performing model. Martens are considered deep-wood specialists (Buskirk and Powell 1994), and thus the strong negative effect of developed land cover on genetic connectivity is expected. In addition, previous studies have highlighted the effect of elevation on gene flow in mountainous regions of Pacific marten (*M. caurina*) distribution (Wasserman et al. 2010).

Genetic drift may represent another major influence on observed genetic distances in our study area. The observed subpopulations are believed to have become isolated during the late nineteenth or early twentieth centuries, and likely persisted for several generations in isolated relicts with low population sizes (Aylward et al. 2019). Random loss of alleles in isolated relicts is believed to have occurred, which may heterogeneously inflate the observed genetic distance between local populations in our study area (Richardson et al. 2016). Indeed, the effects of genetic drift in subpopulations have been estimated to contribute up to 41% of the variance in genetic distance (*T*) in empirical data sets (Prunier et al. 2017a).

Landscape connectivity during population declines in the 1800s may explain some variance in genetic distance that is unexplained by current landscape conditions. Historical landscape data are not available at the appropriate scale and resolution to investigate these effects. Based on estimates of T in this study, landscape predictors in our models contributed up to 17.9% of the total variance in genetic distance (range = 4.2–17.9; Table 1). These numbers are within the typical range of previously published landscape genetics studies using CA with microsatellite data (Renner et al. 2016; Prunier et al. 2017b); however, the majority of variance in genetic distance remains unexplained (Table 2).

**Table 2:** Parameter values and 95% confidence intervals for predictor landscape variables in resistance surfaces for each study area.

Study area	Variable	*R*_max_	*β*	rs	*U*	*T*
Regional						
NE	For	10	0.067 (0.028, 0.106)	0.860 (0.816, 0.897)	0.4 (0.2, 0.9)	6.8
	Elev	10	0.023 (0.015, 0.031)	0.840 (0.791, 0.881)	1.2 (0.7, 1.8)	6.5
	Dev	500	0.188 (0.145, 0.229)	0.616 (0.536, 0.688)	1.2 (0.7, 1.8)	3.5
	Slope	2	0.461 (0.142, 0.751)	0.335 (0.238, 0.427)	0.1 (0.0, 0.3)	1.1
Subregional						
NY	Dev	500	0.279 (0.184, 0.371)	0.562 (0.225, 0.799)	2.1 (0.5, 4.8)	1.6
	Slope	10	−0.241 (−0.321, −0.160)	−0.740 (−0.925, −0.485)	2.9 (0.8, 5.4)	2.7
NHME	Elev	2	0.108 (0.002, 0.230)	0.850 (0.744, 0.906)	0.7 (0.1, 1.5)	5.5
	Dev	10	0.335 (0.078, 0.580)	0.201 (0.034, 0.329)	0.6 (0.1, 1.6)	0.3
	Slope	10	0.206 (0.127, 0.276)	0.317 (0.164, 0.435)	0.4 (0.1, 1.0)	0.8
Local						
NY-W	Dev	50	0.850 (0.521, 1.179)	0.714 (0.487, 0.892)	7.7 (3.6, 12.0)	6.0
	Slope	500	−0.005 (−0.007, −0.003)	−0.583 (−0.794, −0.275)	5.6 (2.3, 12.2)	4.0
NH	Temp	2	0.848 (0.534, 1.162)	0.925 (0.804, 0.990)	3.3 (1.3, 6.2)	5.4
	Slope	2	0.960 (0.204, 1.716)	0.670 (0.444, 0.811)	0.7 (0.0, 1.7)	2.8

Covariates tested in landscape genetics models were forest land cover (For), spruce–fir land cover (SF), winter temperature (Temp), elevation (Elev), developed land cover (Dev), road density (Roads), and slope (Slope). Landscape genetics models were parameterized by maximum-likelihood population effects (MLPE; regional and subregional study areas) or multiple regression of distance matrices (MRDM; local study areas). *β* values are the coefficients of independent variables parameterized by MLPE or MRDM. rs values are structure coefficients estimated by CA. *U* values are the unique contribution (%) of each predictor to the total variance in genetic distance. *T* values are the estimated total contribution of predictors to the variance in genetic distance (i.e., the sum of unique and shared contributions).

### Management implications

Although vegetation conditions in the region are improving for forest carnivores such as martens (Foster et al. 2002), climate conditions and increasing development may allow larger carnivores such as red fox (*Vulpes vulpes*), coyote (*Canis latrans*), and fisher (*Pekania pennanti*), to outcompete martens (Carroll 2007; Sirén et al. 2017). Climate change is predicted to decrease demographic potential and contract the range of martens in the northeastern US (Carroll 2007). Our results show that a warming climate could also decrease gene flow, a pattern also observed in Canada lynx (*Lynx canadensis*) at their southern periphery (Koen et al. 2014b). Furthermore, the regional corridor map indicated complete avoidance of developed areas (Fig. 2). Future expansion of urban and residential areas would likely have adverse effects on genetic connectivity of marten populations in the northeastern US.

### Replication in landscape genetics

Landscape genetics is a developing field with a broad array of methodological approaches and study design considerations (Richardson et al. 2016). Previous studies have shown that results from different portions of species’ ranges do not necessarily align (Short Bull et al. 2011; Castillo et al. 2016). Our study further demonstrates that the choice of study location, spatial scale, and modeling technique can yield different results across subsets of a study system. We also showed that replicating study sites across multiple scales can help elucidate where potential sampling biases are affecting landscape-genetic inferences. These are important results to highlight from a practical perspective, as conclusions drawn from just one of the sites in our study area could potentially encourage management strategies that appear counterproductive in other sites within the region.

## Supplementary information

Supplemental Information

## Data Availability

The genetic data used in this study are available through the Dryad Digital Repository, 10.5061/dryad.p8cz8w9kr.
