# Supplementary material for: Multiscale landscape genetics of American marten at their southern range periphery
Source: Heredity (Edinb). 2020 Jan 28;124(4):550–61. doi: 10.1038/s41437-020-0295-y (PMC7080830; doi:10.1038/s41437-020-0295-y)
Supplement: Supplementary file 1 — Supplemental Information [file 41437_2020_295_MOESM1_ESM.pdf]

Supplemental Information

Supplemental Information I

Sources of geospatial data used in the analysis.

Covariate	Original Resolution (m)	Units	US Data Source	Canada Data Source
Development	250 x 250	Percent cover	National Land Cover Database (NLCD)	MODIS Land Cover (Canadian Centre for Remote Sensing)
Elevation	30 x 30	Meters	National Elevation Dataset (NED)	Canadian Digital Elevation Model (CDEM)
Forest	250 x 250	Percent cover	National Land Cover Database (NLCD)	Natural Resources Canada (Treed Land Cover)
Occupancy	250 x 250	Probability	Aylward et al. 2018	Aylward et al. 2018
Roads	250 x 250	km/ sq. km	State Departments of Transportation (ME, NH, VT, NY)	Canadian National Road Network
Spruce-Fir	250 x 250	Percent cover	USGS GAP Analysis Program	Natural Resources Canada (knn Genus)
Slope	30 x 30	Degrees	Derived from NED	Derived from CDEM
Temperature	800 x 800	Degrees (C)	WorldClim Global Climate Data	WorldClim Global Climate Data

Supplemental Information II

Covariates, R_{max} values tested, and optimal R_{max} values in each study area based on AICc (NE, NY, NHME) or Mantel r (NYW, NYE, NH, ME). NE = regional study area, NY = New York subregion, NHME = northern New England subregion, NYW = western New York local study area, NYE = eastern New York local study area, NH = New Hampshire local study area, ME = Maine local study area. IBD = isolation by distance.

Landscape Variable	Rmax tested	Optimal Rmax						
		NE	NY	NHME	NYW	NYE	NH	ME
IBD	-	-	-	-	-	-	-	-
Occupancy	2, 5, 10, 25, 50, 100, 200, 500	5	2	2	500	2	2	5
Forest	2, 5, 10, 25, 50, 100, 200, 500	10	50	2	2	2	200	500
Spruce-Fir	2, 5, 10, 25, 50, 100, 200, 500	5	2	2	2	2	2	2
Elevation	2, 5, 10, 25, 50, 100, 200, 500	10	2	2	500	500	2	500
Temperature	2, 5, 10, 25, 50, 100, 200, 500	10	2	2	500	500	2	5
Roads	2, 5, 10, 25, 50, 100, 200, 500	10	2	2	500	2	500	10
Development	2, 5, 10, 25, 50, 100, 200, 500	500	500	10	50	500	5	25
Slope	2, 5, 10, 25, 50, 100, 200, 500	2	10	10	500	500	2	2

Supplemental Information III

Multivariate model set and AICc scores. Orange fill indicates a model that was removed from consideration due to highly correlated variables (VIF > 5). Red shading indicates a model that was removed from consideration due to one or more uninformative covariates (coefficient 95% confidence interval included 0). Green shading indicates the model that was selected as the resistance surface for the study area. NE = regional study area, NY = New York subregion, NHME = northern New England subregion, NYW = western New York local study area, NYE = eastern New York local study area, NH = New Hampshire local study area, ME = Maine local study area.

Model	AICc						
	NE	NY	NHME	NYW	NYE	NH	ME
IBD	3674.31	637.85	1184.42	269.00	68.23	478.40	74.39
Occ	3660.15	646.66	1215.06	260.35	70.31	479.48	74.69
For	3644.22	645.20	1207.60	262.72	70.05	489.45	74.99
SF	3654.29	649.41	1182.67	269.97	70.00	465.46	75.62
Elev	3653.96	649.47	1188.41	269.92	66.06	472.31	76.20
Temp	3647.01	650.26	1194.63	268.83	65.94	449.14	76.53
Roads	3682.98	638.86	1215.83	254.68	69.49	493.53	76.57
Dev	3680.75	631.11	1214.01	251.70	68.14	480.01	76.59
Slope	3777.51	627.88	1169.24	258.01	68.98	470.61	76.65
For + Elev	3607.06	643.66	1189.17	264.20	67.88	474.25	76.78
For + Temp	3602.19	646.26	1196.74	264.77	67.63	448.40	76.99
For + Roads	3634.87	643.97	1207.70	256.72	71.54	489.44	77.07
For + Dev	3585.21	638.66	1208.90	253.00	69.31	481.92	77.09
For + Slope	3607.80	629.74	1169.23	254.92	68.96	466.60	77.09
SF + Elev	3656.32	651.45	1186.59	271.98	67.99	466.99	77.30
SF + Temp	3651.83	646.85	1185.92	268.16	67.39	447.98	77.42
SF + Roads	3644.35	632.57	1168.59	255.69	71.50	457.95	77.46
SF + Dev	3538.14	630.59	1184.45	253.74	70.21	464.99	77.61
SF + Slope	3646.49	630.83	1163.56	260.06	70.83	455.95	77.68
Elev + Roads	3643.92	626.48	1180.37	253.42	67.32	467.31	77.75
Elev + Dev	3535.99	628.36	1188.36	253.47	68.23	470.25	78.22
Elev + Slope	3651.11	631.78	1168.46	259.76	67.28	462.61	78.25
Temp + Roads	3639.62	633.96	1190.86	256.02	68.08	440.32	78.47
Temp + Dev	3547.27	631.40	1196.41	253.75	67.36	450.89	78.61
Temp + Slope	3643.55	631.19	1167.22	257.97	68.11	444.94	78.62
Roads + Slope	3648.72	617.96	1168.49	248.35	69.73	472.17	78.68
Dev + Slope	3608.90	607.53	1163.50	235.04	68.40	461.34	78.68
For + Elev + Roads	3612.30	634.93	1182.26	255.48	69.49	468.97	78.69
For + Elev + Dev	3535.13	634.65	1183.45	254.14	69.63	469.61	78.69
For + Elev + Slope	3599.27	631.63	1170.22	256.67	69.47	463.97	78.78
For + Temp + Roads	3607.30	641.41	1192.10	258.09	68.70	442.02	79.06
For + Temp + Dev	3541.38	638.20	1198.07	254.90	69.57	448.01	79.09
For + Temp + Slope	3594.13	633.21	1169.32	256.82	69.37	446.26	79.20
For + Roads + Slope	3605.33	626.55	1169.42	250.35	71.13	468.33	79.21
For + Dev + Slope	3547.41	617.31	1165.96	237.04	70.61	463.18	79.26
SF + Elev + Roads	3649.98	626.90	1172.59	251.38	68.96	459.75	79.33
SF + Elev + Dev	3542.02	630.46	1188.43	254.26	70.21	466.49	79.33
SF + Elev + Slope	3653.06	628.58	1165.04	260.13	69.27	456.02	79.43
SF + Temp + Roads	3646.36	633.89	1172.12	257.66	69.57	441.30	79.44
SF + Temp + Dev	3545.72	631.42	1187.11	255.76	68.87	449.79	79.46
SF + Temp + Slope	3648.06	596.73	1166.83	240.30	69.54	444.71	79.58
SF + Roads + Slope	3633.88	612.28	1158.67	248.86	71.86	454.34	79.58
SF + Dev + Slope	3530.57	607.65	1162.94	236.41	70.55	454.75	79.62
Elev + Roads + Slope	3638.08	611.14	1167.53	247.97	69.13	462.85	79.68
Elev + Dev + Slope	3533.38	608.03	1165.91	236.47	69.37	459.39	79.78
Temp + Roads + Slope	3632.78	618.80	1166.61	250.29	70.25	440.79	80.62
Temp + Dev + Slope	3541.38	610.77	1165.90	237.10	69.37	446.20	80.79
For + Elev + Roads + Slope	3604.37	620.72	1169.36	250.05	70.61	463.56	80.82
For + Elev + Dev + Slope	3530.24	617.43	1163.37	238.55	71.60	461.18	80.89
For + Temp + Roads + Slope	3599.23	627.89	1168.55	252.34	70.02	442.74	81.26
For + Temp + Dev + Slope	3533.12	620.43	1166.59	239.10	71.56	446.15	81.28
SF + Elev + Roads + Slope	3642.93	613.28	1160.65	249.39	70.93	455.17	81.39
SF + Elev + Dev + Slope	3538.31	609.41	1165.29	238.49	71.47	454.69	81.52
SF + Temp + Roads + Slope	3639.53	593.61	1162.16	241.17	71.79	441.83	81.61
SF + Temp + Dev + Slope	3539.11	585.95	1165.32	229.50	71.11	446.12	81.61

Supplemental Information IV

Statistical summaries of variable availability in study areas. Asterisk (*) indicates a study area where the variable was present in the final resistance surface.

Developed Land Cover						Roads					
Study Site	MIN	MAX	RANGE	MEAN	STD	Study Site	MIN	MAX	RANGE	MEAN	STD
Regional*	0	100.00	100.00	2.22	9.71	Regional	0	23.00	23.00	0.96	1.23
NY*	0	66.67	66.67	0.27	2.62	NY	0	9.34	9.34	0.41	0.67
NHME*	0	77.78	77.78	0.56	3.66	NHME	0	13.06	13.06	0.42	0.65
NYW*	0	33.33	33.33	0.05	0.92	NYW	0	4.55	4.55	0.36	0.57
NH	0	77.78	77.78	2.02	7.06	NH	0	13.06	13.06	0.75	0.94
NYE	0	66.67	66.67	0.62	4.19	NYE	0	9.34	9.34	0.49	0.83
ME	0	33.33	33.33	0.21	1.79	ME	0	4.26	4.26	0.23	0.39
Elevation						Spruce-Fir Land Cover					
Study Site	MIN	MAX	RANGE	MEAN	STD	Study Site	MIN	MAX	RANGE	MEAN	STD
Regional*	0	1841	1841	289.50	197.74	Regional	0	100.00	100.00	20.30	21.86
NY	255	1516	1261	598.92	137.58	NY	0	98.00	98.00	13.81	17.35
NHME*	62	1841	1779	404.07	178.90	NHME	0	100.00	100.00	40.44	21.69
NYW	371	1108	737	601.55	104.50	NYW	0	87.00	87.00	9.53	10.63
NH	161	1841	1680	523.22	207.93	NH	0	100.00	100.00	32.93	21.64
NYE	255	1516	1261	621.62	194.26	NYE	0	98.00	98.00	23.36	24.94
ME	93	1439	1346	303.42	135.40	ME	5.00	99.00	94.00	44.81	17.29
Forest Land Cover						Slope					
Study Site	MIN	MAX	RANGE	MEAN	STD	Study Site	MIN	MAX	RANGE	MEAN	STD
Regional*	0	100	100	79.58	28.08	Regional*	0.00	20.00	20.00	1.77	1.89
NY	0	100	100	89.84	15.59	NY*	0.02	16.14	16.12	2.59	2.30
NHME	0	100	100	89.25	17.96	NHME*	0.00	20.00	20.00	2.42	2.26
NYW	0	100	100	89.19	16.18	NYW*	0.02	11.38	11.36	1.87	1.57
NH	0	100	100	92.47	14.29	NH*	0.01	17.92	17.91	3.88	2.91
NYE	22.22	100	77.78	91.99	12.57	NYE	0.05	16.14	16.09	3.91	2.91
ME	11.11	100	88.89	93.17	12.55	ME	0.00	20.00	20.00	1.70	1.89
Occupancy						Temperature					
Study Site	MIN	MAX	RANGE	MEAN	STD	Study Site	MIN	MAX	RANGE	MEAN	STD
Regional	0.00	98.96	98.96	49.88	32.32	Regional	-5.30	5.90	11.20	0.17	2.12
NH	0.36	98.96	98.60	69.05	25.35	NY	-3.64	2.60	6.24	0.08	0.78
NHME	0.36	98.96	98.60	76.44	20.42	NHME	-5.30	2.78	8.08	-1.22	1.32
NY	4.84	98.92	94.08	78.57	18.54	NYW	-1.70	1.38	3.08	-0.05	0.44
NYW	11.40	97.92	86.52	80.60	15.78	NH*	-4.46	2.40	6.86	-0.26	1.06
NYE	4.84	98.92	94.08	77.12	20.80	NYE	-3.64	2.60	6.24	0.16	1.14
ME	9.72	98.44	88.72	85.07	13.13	ME	-5.30	0.38	5.68	-1.78	0.95

Supplemental Information V

Explanatory power of top-scoring resistance models for each study site in comparison to isolation-by-distance (IBD) models that do not incorporate landscape effects.

Study Area	Model	AICc	T
Regional			
NE	For + Elev + Dev + Slope	3530.24	17.9
	IBD	3674.31	6.1
Subregional			
NY	Dev + Slope	607.53	4.2
	IBD	637.85	1.3
NHME	Elev + Dev + Slope	1165.91	6.6
	IBD	1184.42	5.5
Local			
NYW	Dev + Slope	235.04	10.0
	IBD	269.00	0.4
NH	Temp + Slope	444.94	8.0
	IBD	478.40	1.8

Supplemental Information VI

Analysis of the regional results with VTS removed or included. Models that contributed to the 99% of cumulative AICc weight are shown, along with metrics from the commonality analysis. The model selected to represent the resistance surface, based on the model with the greatest T, does not change based on whether VTS samples are included in the analysis.

VTS Removed						
Model (AICc)	Variable	rs	U	C	T	T (model)
FEDS (3530.24)	For	0.860 (0.816, 0.900)	0.004	0.064	0.068	0.179*
	Elev	0.840 (0.786, 0.879)	0.012	0.053	0.065	
	Dev	0.616 (0.542, 0.676)	0.012	0.023	0.035	
	Slope	0.335 (0.253, 0.404)	0.001	0.010	0.011	
SfDS (3530.57)	SF	0.874 (0.816, 0.910)	0.039	0.022	0.061	0.106
	Dev	0.659 (0.588, 0.730)	0.018	0.017	0.035	
	Slp	0.359 (0.256, 0.470)	0.000	0.010	0.010	
FTDS (3533.12)	For	0.899 (0.847, 0.930)	0.011	0.056	0.067	0.169
	Temp	0.825 (0.772, 0.865)	0.004	0.053	0.057	
	Dev	0.644 (0.575, 0.708)	0.008	0.027	0.035	
	Slp	0.351 (0.256, 0.442)	0.001	0.009	0.010	
EDS (3533.38)	Elev	0.859 (0.802, 0.901)	0.047	0.018	0.065	0.110
	Dev	0.630 (0.549, 0.706)	0.022	0.013	0.035	
	Slp	0.343 (0.269, 0.426)	0.000	0.010	0.010	
FED (3535.13)	For	0.863 (0.818, 0.900)	0.004	0.064	0.068	0.168
	Elev	0.842 (0.791, 0.891)	0.015	0.05	0.065	
	Dev	0.618 (0.530, 0.690)	0.013	0.022	0.035	
ED (3535.99)	Elev	0.858 (0.803, 0.894)	0.053	0.012	0.065	0.100
	Dev	0.629 (0.554, 0.703)	0.023	0.012	0.035	
SfD (3538.14)	SF	0.874 (0.817, 0.907)	0.045	0.016	0.061	0.096
	Dev	0.658 (0.594, 0.741)	0.019	0.016	0.035	
VTS included						
Model (AICc)	Variable	rs	U	C	T	T (model)
FTDS (4244.36)	For	0.924 (0.882, 0.949)	0.017	0.04	0.057	0.131
	Temp	0.737 (0.667, 0.788)	0.002	0.037	0.039	
	Dev	0.646 (0.568, 0.708)	0.006	0.024	0.030	
	Slp	0.256 (0.168, 0.340)	0.000	0.005	0.005	
FEDS (4245.06)	For	0.884 (0.835, 0.919)	0.006	0.055	0.061	0.148*
	Elev	0.817 (0.765, 0.859)	0.008	0.044	0.052	
	Dev	0.619 (0.548, 0.691)	0.009	0.021	0.030	
	Slp	0.245 (0.160, 0.339)	0.000	0.005	0.005	
FED (4247.75)	For	0.884 (0.843, 0.923)	0.006	0.055	0.061	0.143
	Elev	0.817 (0.749, 0.866)	0.010	0.042	0.052	
	Dev	0.619 (0.548, 0.692)	0.010	0.020	0.030	
FTD (4250.09)	For	0.925 (0.886, 0.954)	0.018	0.043	0.061	0.129
	Temp	0.737 (0.680, 0.795)	0.003	0.036	0.039	
	Dev	0.647 (0.572, 0.715)	0.006	0.023	0.029	
SfDS (4250.80)	SF	0.867 (0.814, 0.907)	0.033	0.016	0.049	0.085
	Dev	0.677 (0.599, 0.741)	0.016	0.016	0.032	
	Slp	0.268 (0.154, 0.353)	0.000	0.004	0.004	
ED (4253.55)	Elev	0.851 (0.799, 0.897)	0.042	0.010	0.052	0.082
	Dev	0.645 (0.569, 0.712)	0.020	0.010	0.030	
